# Psychiatric disorders, psychotropic medication use and falls among women: an observational study

**DOI:** 10.1186/s12888-015-0439-4

**Published:** 2015-04-08

**Authors:** Lana J Williams, Julie A Pasco, Amanda L Stuart, Felice N Jacka, Sharon L Brennan, Amelia G Dobbins, Risto Honkanen, Heli Koivumaa-Honkanen, Päivi H Rauma, Michael Berk

**Affiliations:** 1 IMPACT Strategic Research Centre, School of Medicine, Deakin University, Barwon Health, PO Box 281 (Barwon Health), Geelong, 3220 Australia; 2 NorthWest Academic Centre, Department of Medicine, The University of Melbourne, Western Health, St Albans, Australia; 3 Department of Psychiatry, The University of Melbourne, Parkville, Australia; 4 Australian Institute for Musculoskeletal Sciences, Melbourne, Australia; 5 Bone and Cartilage Research Unit, Surgery, Institute of Clinical Medicine, University of Eastern Finland, Kuopio, Finland; 6 Institute of Clinical Medicine, Psychiatry, University of Eastern Finland, Joensuu, Finland; 7 Department of Psychiatry, Kuopio University Hospital, Kuopio, Finland; 8 Department of Psychiatry, South-Savonia Hospital District, Mikkeli, Finland; 9 Department of Psychiatry, North Karelia Central Hospital, Joensuu, Finland; 10 Department of Psychiatry, SOSTERI, Savonlinna, Finland; 11 Department of Psychiatry, SOTE, Iisalmi, Finland; 12 Department of Psychiatry, Lapland Hospital District, Rovaniemi, Finland; 13 Department of Social Pharmacy, Faculty of Health Sciences, University of Eastern Finland, Finland, Finland; 14 The Florey Institute of Neuroscience and Mental Health, The University of Melbourne, Parkville, Australia; 15 Orygen, The National Centre of Excellence in Youth Mental Health, The University of Melbourne, Parkville, Australia

**Keywords:** Depression, Anxiety, Falls, Psychotropic medication, Antidepressants, Benzodiazepine

## Abstract

**Background:**

Psychotropic agents known to cause sedation are associated with an increased risk of falls, but the role of psychiatric illness as an independent risk factor for falls is not clear. Thus, this study aimed to investigate the association between psychiatric disorders, psychotropic medication use and falls risk.

**Methods:**

This study examined data collected from 1062 women aged 20-93 yr (median 50 yr) participating in the Geelong Osteoporosis Study, a large, ongoing, population-based study. Depressive and anxiety disorders for the preceding 12-month period were ascertained by clinical interview. Current medication use and falls history were self-reported. Participants were classified as fallers if they had fallen to the ground at least twice during the same 12-month period. Anthropometry, demographic, medical and lifestyle factors were determined. Logistic regression was used to test the associations, after adjusting for potential confounders.

**Results:**

Fifty-six women (5.3%) were classified as fallers. Those meeting criteria for depression within the past 12 months had a 2.4-fold increased odds of falling (unadjusted OR = 2.4, 95% CI 1.2-4.5). Adjustment for age and mobility strengthened the relationship (adjusted OR = 2.7, 95% CI 1.4-5.2) between depression and falling, with results remaining unchanged following further adjustment for psychotropic medication use (adjusted OR = 2.7, 95% CI 1.3-5.6). In contrast, past (prior to 12-month) depression were not associated with falls. No association was observed between anxiety and falls risk. Falling was associated with psychotropic medication use (unadjusted OR = 2.8, 95% CI 1.5-5.2), as well as antidepressant (unadjusted OR = 2.4, 95% CI 1.2-4.8) and benzodiazepine use (unadjusted OR = 3.4, 95% CI 1.6-7.3); associations remained unchanged following adjustment for potential confounders.

**Conclusion:**

The likelihood of falls was increased among those with depression within the past 12 months, independent of psychotropic medication use and other recognised confounders, suggesting an independent effect of depression on falls risk. Psychotropic drug use was also confirmed as an independent risk factor for falls, but anxiety disorders were not. Further research into the underlying mechanisms is warranted.

## Background

Falls are common, with up to 30% of community dwelling and 50% of institutionalized older adults falling every year [1]. It has been reported that as many as 10% of falls result in major injury often leading to institutionalization and death. In Australia alone, there has been a four-fold increase in the number of deaths resulting from falls since 2002; this is thought to be due to the aging population, with women accounting for over half of deaths due to falling [2].

Advanced age, mobility, sensory and medical factors, cognitive capacity, certain medications, and environmental hazards are known risk factors for falls among older adults [3]. Another possible risk factor for falls is depression. It has been shown to precede falls in the elderly, and is also a possible outcome of falls, as with increased anxiety, decreased satisfaction with life and activity restriction [3,4]. Psychotropic medications also increase risk of falls [5], possibly due to their sedative nature, and thus are a potential mediator of the relationship between psychiatric disorders and falls. However, the exact underlying mechanism(s) of action for the relationship between psychiatric disorders and falls remains unclear. Moreover, it is uncertain whether the relationship occurs among younger adults or those residing within the community, rather than in institutions.

As such, we aimed to investigate the association between depressive and anxiety disorders, psychotropic medication use, and falls in a large, randomly-selected, population-based sample of women spanning the full adult age-spectrum. We also aimed to examine the role of potential confounders in accounting for any observed associations.

## Methods

### Participants

Data were derived from an age-stratified, population-based sample of women enrolled in the Geelong Osteoporosis Study (GOS). Originally, 1,494 women (aged 20–94 yr, response 77.1%) were randomly recruited from the electoral rolls for the Barwon Statistical Division (south-eastern Australia) between 1994 and 1997 and have returned for ongoing assessment [6]. Between 2004 and 2008, 881 of the original sample returned for a 10-year follow-up assessment (participation 82.1%) and an additional sample of 246 women aged 20–29 years was recruited (participation 70.9%), allowing for continuing investigation of the full adult age range [7]. Of the 1127 women who participated in the GOS during 2004–2008, participants for whom psychiatric data were not available for this phase (n = 32), did not return their questionnaire (n = 10), were unable to recall year of last psychiatric episode (n = 2), and were current users of anticonvulsant (n = 13) and antipsychotic medications (n = 8), were excluded, resulting in a sample of 1062 women aged 20-93 yr eligible for this analysis. All participants gave written, informed consent, and the study was approved by the Human Research Ethics Committee at Barwon Health.

### Measurements

#### Outcome variable

The number of falls occurring during the 12-months prior to the study assessment (2004–2008) was documented by self-report questionnaire. The definition “when you suddenly find yourself on the ground, without intending to get there, after you were in either a lying, sitting or standing position” [8,9] was used to determine a fall. For this analysis, participants were classified as fallers if they had fallen to the ground at least twice during the same 12-month period.

#### Exposure variables

The Structured Clinical Interview for Diagnostic and Statistical Manual of Mental Disorders, Fourth Edition, Non-patient edition (SCID-I/NP) was used to assess lifetime history of depressive disorders, including major depressive disorder (MDD), minor depression, bipolar disorder, dysthymia, mood disorder due to a general medical condition, and substance induced mood disorder and anxiety disorders, including panic disorder, agoraphobia, social phobia, specific phobia, obsessive-compulsive disorder, generalised anxiety disorder, anxiety disorders due to a general medical condition, substance induced anxiety disorder and anxiety disorders not otherwise specified [10]. Participants were classified as having 12-month, past (prior to 12-month), or no history of depressive or anxiety disorders. Trained personnel, with qualifications in psychology, conducted all psychiatric interviews.

Antidepressant and benzodiazepine use was self-reported and was deemed current if used regularly at the time of assessment. Participants were asked to bring in a list of medications or containers to assist with accurate recording of details.

Weight was measured to the nearest 0.1 kg. Information on lifestyle and other health factors was obtained via questionnaire. Mobility was classified as active if vigorous or light exercise was performed regularly, sedentary if normal day to day living is achieved but no appreciable exercise, and limited if activity was restricted to the point of little walking outside of the home, sits in chair or lies in bed most of the time, or bedridden. Use of a walking aid was determined by the question “Do you use a walking aid” (Yes/No). Current health status was measured using a self-report 5-point Likert self-report scale ranging from 1 (Excellent) to 5 (Poor). Alcohol and calcium intake was estimated from a validated food frequency questionnaire [11] and current smoking status (“How many cigarettes do you have each day?”) was documented. Socio-economic status (SES) was ascertained using Socio-Economic Index For Areas (SEIFA) index scores, based on the 2006 Australian Bureau of Statistics Census data; SEIFA scores were used to determine the level of SES via the Index of Relative Socio-economic Advantage and Disadvantage (IRSAD). The IRSAD accounts for parameters measured at the area-level, including high and low income, and type of occupation. A low score using the IRSAD identifies the most disadvantaged (quintile 1), while a high score identifies the most advantaged (quintile 5) [12]. Blood pressure was measured (seated) with a digital meter (A&D Company, model UA-751).

#### Statistics

Differences in characteristics between those with 12 month, past or no history of depression or anxiety were determined using analysis of variance (ANOVA), with Tukey’s Test Statistic for multiple comparisons applied, for continuous variables, Kruskal-Wallis for non-parametric continuous variables, and the chi square test for discrete variables. Post hoc analyses were conducted where appropriate. Logistic regression was used to calculate odds ratios (OR) with 95% confidence interval (95% CI) for falls for those with 12-month and past depression in comparison to those with no depression. The relationship between falls and anxiety disorders (12-month/past/never), were similarly investigated. Covariates included age, weight, psychotropic medication, smoking status, blood pressure, mobility, use of a walking aid, health status, SES, alcohol consumption and calcium intake. These were tested sequentially and only included in the final model if significant. In addition to these covariates, depressive and anxiety disorders were tested when exploring the association between falls and use of any psychotropic medication, antidepressants and benzodiazepines. All interactions were tested. Statistical analyses were performed using Minitab (Version 16; Minitab, State College Pa) and SPSS statistical package 22.0 for Windows (SPSS Inc., Chicago, IL, USA).

## Results

### Depressive disorders and falls

One hundred and forty six (13.8%) women were classified as having 12-month depression, 148 (13.9%) women had past depression, and 768 (72.3%) had no history of depression. The groups differed in age, weight, smoking status, diastolic blood pressure, walking aid use, health status, falls history and psychotropic use; otherwise the groups were similar (Table 1). Those meeting criteria for 12-month depression had a 2.4-fold increased odds of falling compared to those with no history of depression (unadjusted OR = 2.4, 95% CI 1.2-4.5, p = 0.01). Adjustment for age and mobility strengthened the relationship (adjusted OR = 2.7, 95% CI 1.4-5.2, p = 0.004) between depression and falling, with results remaining unchanged following adjustment for psychotropic medication use (adjusted OR = 2.7, 95% CI 1.3-5.6, p = 0.01). Further adjustment for weight, use of walking aid, smoking status, health status, anxiety disorders, blood pressure, SES, alcohol consumption or calcium intake did not affect the association. The odds of falling were not increased among those with past depression (p = 0.35).

**Table 1:** **Characteristics for the whole group, women with 12-month, past and no past depressive or anxiety disorder**

		Depressive disorders				Anxiety disorders			
	All	12 month depression	Past depression	Never	p	12 month anxiety	Past anxiety	Never	p
		n = 146	n = 148	n = 768		n = 99	n = 40	n = 923	
Age (yr)	50.0 (33.5-65.0)	47.0 (33.8-59.0)^a^	46.5 (29.0-59.8)^a^	52.0 (35.0-68.0)	<0.001	44.0 (29.0-56.0)^b^	54.0 (32.8-64.8)	51.0 (34.0-67.0)	0.008
Weight (kg)	69.4 (61.4-80.8)	72.9 (63.4-82.5)^a^	71.7 (62.8-79.9)	68.3 (60.9-80.3)	0.03	72.0 (63.4-80.5)	68.7 (60.4-80.2)	69.0 (61.2-81.0)	0.40
Smoking (current)	149 (14.0%)	33 (22.6%)^a^	22 (14.9%)	94 (12.2%)	0.004	21 (21.2%)	6 (15.0%)	122 (13.2%)	0.09
Systolic BP (mmHg)	125.1 ± 17.5	124.2 ± 17.0	123.5 ± 14.7	125.5 ± 18.0	0.41	122.2 ± 17.8	126.7 ± 16.8	125.0 ± 17.4	0.25
Diastolic BP (mmHg)	75.7 ± 11.0	78.0 ± 10.9^a^	74.9 ± 9.9	75.4 ± 11.1	0.03	76.1 ± 11.5	76.8 ± 10.7	75.6 ± 10.9	0.75
Mobility (current)					0.10				0.36
Active	831 (78.4%)	107 (73.3%)	123 (83.7%)	601 (78.4%)		74 (74.8%)	34 (85.0%)	723 (78.5%)	
Sedentary	174 (16.4%)	32 (21.9%)	21 (14.3%)	121 (15.8%)		21 (21.2%)	3 (7.5%)	150 (16.3%)	
Limited	55 (5.2%)	7 (5.3%)	3 (2.3%)	45 (5.9%)		4 (4.0%)	3 (7.5%)	48 (5.2%)	
Walking aid (current)	58 (5.5%)	5 (3.4%)^a^	3 (2.0%)^a^	50 (6.5%)	0.05	5 (5.1%)	1 (2.5%)	52 (5.7%)	0.68
Health status (current)					<0.001				0.04
Excellent-Very good	621 (58.6%)	64 (43.8%)^a^	83 (56.9%)	474 (61.8%)		48 (48.5%) ^b^	20 (50.0%)	553 (60.1%)	
Good	322 (30.4%)	52 (35.6%)	50 (34.3%)	220 (28.7%)		36 (36.4%)^b^	18 (45.0%)^b^	268 (29.1%)	
Fair-Poor	116 (11.0%)	30 (20.6%)^a^	13 (8.9%)	73 (9.5%)		15 (15.2%)	2 (5.0%)^b^	99 (10.8%)	
Alcohol intake (g/d)	2.9 (0.4-12.1)	3.1 (0.4-14.8)	3.9 (0.5-14.7)	2.6 (0.3-10.6)	0.06	2.5 (0.3-13.5)	4.7 (0.4-12.4)	2.8 (0.4-11.7)	0.99
Calcium intake (mg/d)	836.9(607.6-1070.2)	836.1(556.6-1098.5)	794.2(572.6-1045.3)	840.6(617.6-1072.0)	0.43	787.8(549.2-1099.4)	920.1(676.2-1208.9)	836.3(608.1-1065.2)	0.32
Falls (2 or more)	56 (5.3%)	14 (9.6%)^a^	9 (6.1%)	33 (4.3%)	0.03	9 (9.1%)	4 (10.0%)	43 (4.7%)	0.07
Socioeconomic status					0.70				0.89
Quintile 1 (most disadvantaged)	165 (15.4%)	28 (19.2%)	22 (14.9%)	115 (15.0%)		17 (17.2%)	7 (17.5%)	141 (15.3%)	
Quintile 2	229 (21.6%)	32 (21.9%)	30 (20.3%)	167 (21.7%)		23 (23.2%)	6 (15.0%)	200 (21.7%)	
Quintile 3	242 (22.8%)	32 (21.9%)	34 (23.0%)	176 (22.9%)		19 (19.2%)	11 (27.5%)	212 (23.0%)	
Quintile 4	210 (19.8%)	31 (21.2%)	25 (16.9%)	154 (20.1%)		22 (22.2%)	6 (15.0%)	182 (19.7%)	
Quintile 5	216 (20.3%)	23 (15.8%)	37 (25.0%)	156 (20.3%)		18 (18.2%)	10 (25.0%)	188 (20.4%)	
Psychotropic use (current)	159 (15.0%)	59 (40.4%)^a^	33 (23.3%)^a^	67 (8.7%)	<0.001	29 (29.3%)^b^	16 (40.0%)^b^	114 (12.4%)	<0.001
benzodiazepine use	64 (6.0%)	16 (11.0%)^a^	9 (6.1%)	39 (5.1%)	0.02	13 (13.1%)^b^	5 (12.5%)^b^	46 (5.0%)	0.001
antidepressant use	120 (11.3%)	54 (37.0%)^a^	27 (18.2%)^a^	39 (5.1%)	<0.001	23 (23.2%)^b^	15 (37.5%)^b^	82 (8.9%)	<0.001

^a^significantly different from no lifetime history of depressive disorders (never).

^b^significantly different from no lifetime history of anxiety disorders (never).

Values are given as mean (± SD), median (IQR) or n (%).

### Anxiety disorders and falls

Ninety-nine (9.3%) women were classified as having a 12-month anxiety disorder, a further 40 (3.8%) had suffered from an anxiety disorder in the past, and 923 (86.9%) had no previous history. The groups differed in terms of age, health status and psychotropic medication use; otherwise the groups were similar (Table 1). Both 12-month (p = 0.06) and past (p = 0.14) anxiety disorders were not associated with an increased risk of falling.

### Psychotropic medication use and falls

One hundred and fifty nine (15.0%) women reported psychotropic medication use, with 64 (6.0%) reporting benzodiazepine use and 120 (11.3%) antidepressant use. Any psychotropic medication use (unadjusted OR = 2.9, 95% CI 1.6-5.2, p ≤ 0.001), antidepressant use (unadjusted OR = 2.5, 95% CI 1.3-4.9, p = 0.005), and benzodiazepine use (unadjusted OR = 3.3, 95% CI 1.5-7.1, p = 0.002) were each associated with falling. Adjustment for age, weight, mobility, use of walking aid, smoking status, health status, depressive and anxiety disorders, blood pressure, SES, alcohol consumption or calcium intake did not explain the relationships.

## Discussion

The findings of this cross-sectional, population-based study across the adult age spectrum showed that women meeting criteria for 12-month depression had an increased risk of falling compared to those with no history of depression independent of anthropometric, demographic, medical and lifestyle factors. Psychotropic use was also confirmed as an independent risk factor for falls; but, interestingly neither past depression nor anxiety disorders were associated with falling.

These findings are concordant with recent meta-analyses of prospective studies reporting depression to be independently associated with increased odds of recurrent falls in older people [1,3]. Studies investigating this relationship in samples of community-dwelling and institutionalised older people have repeatedly reported positive findings. Within a population-based sample of 7,414 elderly women, depressive symptomatology was associated with up to a 40% increased odds of falling, which persisted after adjustment for socio-demographic characteristics, medical conditions, functional status, medication use and other lifestyle factors [13]. Similarly, depressive symptoms, as measured with a 4-item Geriatric Depression Scale, were shown to be associated with recurrent falls in a large group of community dwelling elderly [14]. Utilising a random sample of Medicare claimants (n = 601,922) from Australia, the odds of a fall- related injury was found to be approximately two times greater for elderly men and women with depression [15]. In a prospective cohort study of community-dwelling older people examining predictors of recurrent falls, depression was considered to be as strong a predictor as abnormal postural sway, two or more falls in the previous year, and low scores for hand grip strength [16]. While increased worry and fear of falling in older people has been repeatedly shown [4], the relationship between anxiety disorders and falls is less explored. In contrast to our findings, Whitney et al. found anxiety as measured by the Goldberg Anxiety Scale to be a significant predictor of falling as was poor attention and orientation, increased postural sway with eyes closed, and antidepressant use in a group of cognitively impaired older adults [17]. Another study found the prevalence of anxiety (and depression) to be higher among fallers [18].

These data are consistent with previous findings that psychotropic medication is associated with falls [5]. A recent meta-analysis of 71 studies containing data on risk factors associated with psychotropic drug use among the elderly reported the pooled OR for the association between falls and any psychotropic use to be 1.78 (95% CI 1.57-2.01) [5]. An earlier meta-analysis of studies conducted between 1966 and 1996 reported a similar pooled OR of 1.73 (95% CI, 1.52-1.97) [19]. Both antidepressants and benzodiazepine use alone have been associated with increased falling in the elderly. In the recent aforementioned meta-analysis, antidepressant and benzodiazepine use was associated with a 1.66 (95% CI, 1.4-1.95) and 1.48 (95% CI, 1.23-1.77) fold increased risk of falls, respectively [5]. Dose response relationships are evident, whereby falls rates among nursing home residents have been shown to increase with increasing daily doses of antidepressants [20,21] and benzodiazepines with greater half-lives [22]. Examining the time course of falls to identify specific at-risk periods during antidepressant treatment, Joo *et al.* [23] found of the 104 elderly participants, 40 (38%) fell during the 21 weeks of treatment, with about half (53%) falling during the first six weeks, indicating falls monitoring is warranted during the acute stages of treatment. Similarly, when comparing new and repeat use of benzodiazepines, Maxwell *et al.* [24] reported an increased risk of fall-related hospitalisation for new users of benzodiazepines (OR 2.8, 95% CI 2.2-3.6) and tranquillisers (OR 2.0, 95% CI 1.5-2.6), with risk estimates reducing slightly for repeat users. Other psychotropic agents, including sedatives and hypnotics and antipsychotics, have also been associated with increased falls risk among the elderly [25,26].

Potential mechanistic factors used to explain the relationship between falls and psychiatric disorders include perturbations in gait, functional mobility, cognitive impairment, psychomotor retardation, changes in blood pressure and effects of psychotropic medication [4]. However, in the present study involving individuals spanning the entire adult age range, psychotropic use, mobility, and blood pressure did not significantly influence the relationship between depression and falls. In general, depression has been shown to be associated with changes in gait, including decreases in walking speed, gait unsteadiness, and inability to maintain a stable walking pattern among the elderly [27-29]. In a study of 50 older patients diagnosed with either MDD or bipolar disorder, walking pace tended to be reduced and gait unsteadiness and swing time variability was increased; this predisposes individuals to falls [30].

Postural instability may also play a role in the risk of falls. Within a group of 69 patients admitted to a geriatric hospital unit for ‘spontaneous’ unexplained falls during a 12-month period, impairment in postural abilities in the standing position was shown to differ between the depressed fallers group and non-depressed fallers group [31]. The antidepressants paroxetine and sertraline have been associated with increased falls risk among older adults due to impairments in balance control and body sway [32,33], although another study in older adults found no acute changes in body sway after six weeks of treatment with sertraline [34]. It is noteworthy that the possible risk imposed by gait changes appears to be a state rather than a trait marker of depression, as those with prior but not 12-month depression showed no increase in falls risk.

A major strength of this study is that it examined the relationship between psychiatric disorders and falls within a population-based sample spanning the entire adult age range. Previous research has been limited to the elderly, with the majority of studies involving samples of institutionalised participants. In our models, there was no age interaction, indicating the relationship between depression and falls was similar for young and older women. We recognise that this study has some limitations. Power limitations prevented exploration of the relationship between specific classes of benzodiazepines and antidepressants and falls. Furthermore, we excluded users of other psychotropic agents, including sedatives, hypnotics and antipsychotics, due to the sample size preventing additional subgroup analyses. Medication dose, duration of therapy, and stability are also potential confounders influencing outcomes; however, these variables could not be tested in our multivariate analyses. Likewise, rigorous measures of gait and balance were also not available to be tested. Last, as in all observational studies, there may have been unrecognised confounding.

## Conclusion

In conclusion, there was an increased likelihood of falls among those with 12-month depression, independent of psychotropic medication use and other recognised confounders, suggesting an independent association between depression and falls. Psychotropic use was also confirmed as an independent risk factor for falls, but anxiety disorders were not. The poorer health status observed among women with depression in this study emphasises the disabilities potentially associated with this disorder. Together with data suggesting that one in three community-dwelling individuals over 65 years of age fall each year [35], alongside the high prevalence of depression [7,36], both depression and psychotropic use as risk factors for falls needs to be taken into consideration.

## Abbreviations

ANOVA: Analysis of variance
CI: Confidence Intervals
GOS: Geelong Osteoporosis Study
IRSAD: Index of Relative Socio-economic Advantage and Disadvantage
MDD: Major depressive disorder
OR: Odds ratio
SEIFA: Socio-Economic Index For Areas
SES: Socio-economic status
SCID-I/NP: Structured clinical interview for DSM-IV-TR research version, non-patient edition
